# Virtual Free-Radical Polymerization of Vinyl Monomers in View of Digital Twins

**DOI:** 10.3390/polym15142999

**Published:** 2023-07-10

**Authors:** Elena F. Sheka

**Affiliations:** Institute of Physical Researches and Technology, Peoples’ Friendship University of Russia (RUDN University), 117198 Moscow, Russia; sheka@icp.ac.ru

**Keywords:** chain-reaction mechanism, vinyl monomers, free-radical polymerization, digital twins approach, energy graphs, spin-origin transition state, activation energy

## Abstract

The first case of virtual polymerization based on the concept of digital twins (DTs) is presented. The free-radical polymerization of vinyl monomers is considered to be a chain reaction consisting of a set of elementary ones. Those three types, related to the polymerization initiation and propagation as well as to the termination of polymer chain growth, are discussed. Special sets of DTs, whose total number approaches 60, distinguish each reaction type. The calculations are carried out using a semi-empirical version of the unrestricted Hartree–Fock approximation. The main energy and spin-density parameters of the ground state of the DTs are determined. The barrier profiles of two pairs of DTs are calculated, based on which two Evans–Polanyi–Semenov relations, attributed to elementary reactions of type (1) and (2), are constructed. These provide a quite reliable evaluation of the activation energy for the initiation and propagation of the free-radical polymerization of vinyl monomers in all the cases. The decisive role of spins in the formation of the elementary reaction transition states is established.

## 1. Introduction

Computational chemistry studies of polymers started as classical modeling more than 60 years ago and the field is currently considered as one of the most developed areas of chemical virtuality. Over the years, much progress has been made in polymer modeling, from a microscale view of the topic to intermediate-scale, mesoscale, and macroscopic-scale ones that were basic models for polymers’ quantum chemistry (QC) [1,2,3,4,5,6,7,8,9,10,11,12,13,14,15,16], molecular dynamics [17], coarse-grained [18], and continuum concepts [11,18], respectively. Drastically differing in content, all the concepts, nevertheless, are based on a common view of polymers that are directly modeled as a sequence of points in three-dimensional space. The above reviews and references therein give a reliable idea of the contemporary state of the art in virtual polymer chemistry. Recently, two completely new concepts, stimulated by the ‘big-data treatment’ achievements, have been proposed for application to polymers. These are digital twins (DTs) [19] and quantum computing [20]. Both concepts fundamentally change the modeling paradigm. In the first case, the changing concerns the combination of physical and data-science models to improve the former by incorporating the power of statistics and machine learning. The new simulations receive the status of independent virtual experiments, equal in rights with real ones. In the second case, the system of points in 3D space is replaced by a coloring puzzle according to the Ising model. Quantum computers are extremely efficient at solving problems such as finding the color assignment that satisfies the largest number of ‘given rules’, where each found solution of the optimization problem is associated with a specific polymer configuration.

**Figure 1:**
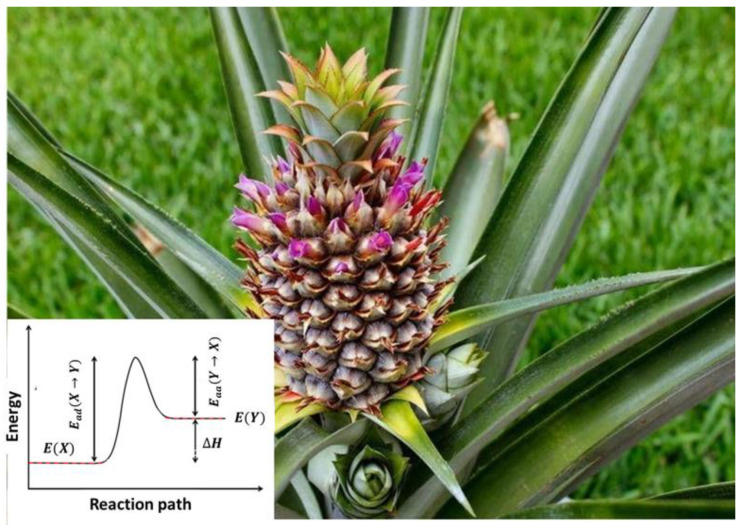
Universal energy graph of the pairwise intermolecular interaction in the system of many possibilities. A photo from a freely accessible collection, pro-dachnikov.com accessed on 1 May 2023, is used for the figure design.

Despite a deep contribution of computational chemistry to polymer science, there are not so many truly resolved issues. The high cost of calculations for models comparable to real systems, the limited capabilities of the available computational software and the lack of complex programs, providing simultaneous consideration of deterministic approaches such as thermodynamics, kinetics, and fluid mechanics, are all undoubtedly reasons for this. However, the exceptional complexity of polymers, which are the final products of multifaceted and multivariate intermolecular interactions, including a large number of participants, is also influential. The fate of a monomer that has fallen into the field of interaction of numerous participants can be figuratively represented as the state of a pineapple on a plant surrounded by sword-like leaves (see Figure 1). Each leaf is a realized path of development in the corresponding direction. Each step on this path is subordinated to a generally common energy graph that might differ in quantity only. In addition to the opportunities used, there are still a large number of others hidden under individual scales of the fruit bark.

The polymer, as a material product, is the result of a choice made among the possible leaves around the fruit and the scales on its bark. Modern computational chemistry is unable to predict this choice. At best, the totality of all approaches, from micro-sized to macro-sized ones, makes it possible to explain the choice made either by Nature or by chemists thanks to their vast experience and intuition. In light of this, the two new concepts of the virtual approach to polymerization have attracted special attention. Actually, as it turns out, the DT concept has allowed the solving of a number of virtual chemistry problems that had not been solved before [21,22]. This circumstance stimulated the author to turn to virtual polymer science from the DT concept viewpoint. It was quite logical for the first testing of this new paradigm to choose an issue that has been well studied experimentally and considered many times virtually by traditional computational chemistry. The free-radical polymerization (FRP) of vinyl polymers is just such an issue.

## 2. Main Elements of Virtual Free-Radical Polymerization

FRP of vinyl monomers [23,24] is at the root of the production of a large percentage of modern polymer products. Generally, the participants in this process are quite numerous and involve monomers, M, free radicals, $R^{•}$, monomer radicals, ${RM}^{•}$, constantly growing oligomer radicals, $RM_{n}^{•}$, and a solvent or some other reaction members. All the participants are connected by the all-with-all intermolecular interaction, which leads to the formation of a large variety of different intermolecular complexes. Thus, the interaction of a monomer with a free radical results in the generation of a monomer radical, the interaction of which with the monomer underlies the growth of oligomer radicals presenting a polymer chain (a polymerization propagation). The interaction of oligomer radicals with each other or with a monomer radical, as well as with free radicals, determines the termination of the polymer chain growth. The interaction of terminated chains determines the formation of the main bulk of the polymer material. The result of each of these elementary processes is greatly influenced by the interaction of their components with solvent molecules [25].

**Scheme 1:**
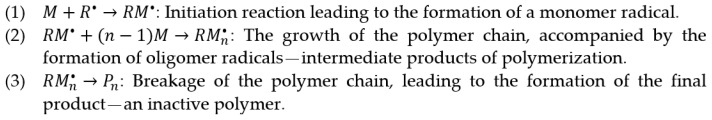
Basic elementary reactions of free-radical polymerization.

Real polymer products are produced in the conditions of the simultaneous implementation of all these events. Evidently, the virtual consideration of the total interactive community seems to be possible within the paradigm of quantum computing, satisfying the largest number of ‘given rules’, only [20]. Nevertheless, about a century ago, theoretical chemistry proposed a way out of this situation by presenting polymerization as a special kind of chain reaction [26,27]. Under these conditions, the polymerization process is determined by a set of elementary reactions [7,28], and the task of theoretical and virtual chemistry is to establish the chemistry of these elementary reactions and find their energetic and kinetic characteristics. In accordance with this, the following basic reactions (in Scheme 1) present FRP’s main features:

Naturally, this elementary-reaction-chain (ERC) approximation does not fully describe the complete polarization process. However, as history has shown, it is a reliable base for the QC analysis of the process, which makes it possible to reveal the mechanisms underlying it. The next steps towards the description of the final product $P_{n}$, as well as its macroscopic properties already go beyond the ERC approximation and require other computational algorithms. Efficient molecular-dynamics algorithms, such as ReaxFF [29,30], have been successfully applied to polymers at the atomistic level. In the present work, the main attention will be focused on the QC consideration of the elementary reactions presented in the scheme.

Traditionally, the thermodynamics of chemical reactions is quite fully described in the framework of transition state theory [28,31] by a standard energy graph (see Figure 1) that includes the total energy of the equilibrium reactant community Y, E(Y), the energy of the X product of the Y pair interaction, $E\left(X\right)$, and the energy of the transition state of the molecular complex under consideration, $E_{TS}\left(X↔Y\right)$. The required values are provided with all available QC software. As for the kinetics, its standard description concerns the rate constant of reactions, $k\left(T\right),$ which is expressed through the Arrhenius equation (Equation (1)).

(1) $$k\left(T\right)=Ae^{\left(\frac{-E_{a}}{RT}\right)}$$

Here, A is a complex frequency factor, while $E_{a}$ represents the activation energy, which is either the energy of the X product’s dissociation, $E_{ad}$, or the Y pair association, $E_{aa}$. There is also one more important energetic parameter that is the reaction enthalpy, ∆H, or coupling energy, $E_{cpl}$ = E(X) − E(Y), which describes either how endothermic or exothermic is the formation of product X from molecular pair Y. The main difficulty in evaluation of the $k\left(T\right)$ value is the highly complicated nature of the frequency factor A. Its determination concerns the basic problems of the rotational–vibrational dynamics of polyatomic molecules, such as a great number of both vibrational and rotational degrees of freedom as well as their anharmonic dynamics, which drastically influences the energetic scheme of the molecules. Computations become highly cumbersome while the obtained results generally differ significantly from empirical data (see reviews [9,10,12,13,14,15] and references therein).

Numerous studies of the kinetics of various reactions have made it possible to reveal a significant simplification of the problem concerning reactions of the same type [28]. As shown, the activation energy, both $E_{ad}$ and $E_{aa}$, is the main factor affecting these reactions’ rates. The feature opens up the possibility of comparing the kinetic behavior of the reactions, such as reactions (1), (2), and (3) in Scheme 1, in terms of this value. Moreover, as it turns out, for these reactions, a linear relationship can be established between the enthalpy of reaction ∆H, or coupling energy $E_{cpl}$, and the activation energy [6,7,13], one of the expressions of which is the Evans–Polanyi–Semenov (EPS) equation, related to $E_{aa}$ in the form

(2) $$E_{aa}=M+\alpha E_{cpl}$$

Here M and α are statistical parameters, approximately constant for a particular set of the same type of reactions while different for other sets. A direct determination of the energy value requires the knowledge of the transition state energy $E_{TS}$. This value can be implemented directly by building barrier profiles of either association of molecular pairs Y or dissociation of X products [32]. However, in the case of elementary reactions related to FRP, the energy $E_{TS}$ is usually evaluated following the approximation of the $E_{tot}\left(X\right)$ by using the synchronous transit-guided quasi-Newton method [13].

In the current paper, the virtual FRP of vinyl monomers is considered in terms of the ERC approximation. The FRP thermodynamics will be presented with standard energy graphs related to sets (1)–(3) of the elementary reactions. The virtual FPR kinetics will be discussed in term of the activation energy $E_{aa}$, or $E_{a}$ below, first obtained from a selected set of barrier profiles to justify the EPS relation (2) and then evaluated by using the latter. The consideration follows the DT concept paradigm.

## 3. Digital Twins Concept, Samples, Virtual Device, and Intellectual Product of Virtual Free-Radical Polymerization of Vinyl Monomers

**Scheme 2:**
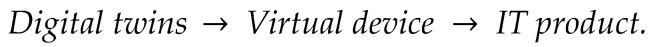
Digital twins concept paradigm.

A general paradigm of the DT concept can be presented schematically (in Scheme 2) as [21,22].

Here, DTs are the molecular models under study, the virtual device is a carrier of a selected software, and the IT product covers a large set of computational results related to the DTs under different actions in the light of the software explored by the virtual device. The quality of the IT product highly depends on how broadly and deeply the designed DTs cover the knowledge concerning the object under consideration and how adequate is the virtual device to peculiarities of this object. The first requirement can be met by a large set of relevant DTs. As for the virtual device, it should not contradict with the object’s nature and should perform calculations that provide reliable IT. Digital twins are completely free from the statistical and random errors that accompany real experiments, and are thus easy to modify. Came into the possession of a virtual device, DTs lay the foundation of a free independent virtual experiment in terms of the process of interest. As for the final IT product, an essential part of which is a comparative analysis of a large number of data produced under the same conditions, it mainly determines trends in the behavior of the object of interest.

**Figure 2:**
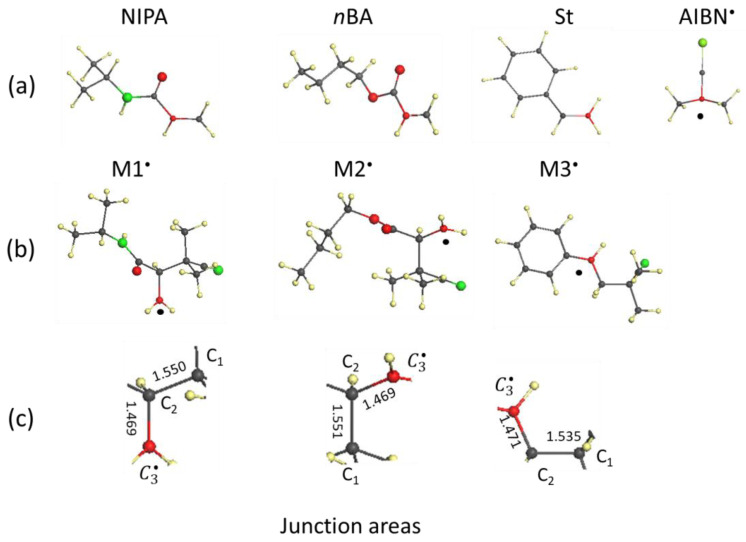
Monomer radicals based on vinyl *sp*^2^C=C bond. Equilibrium structures of monomers *M*1 (NIPA), *M*2 (*n*BA), and *M*3 (St) as well as free radical AIBN^•^ (**a**) and the relevant monomer radicals *RM*1^•^, *RM*2^•^, and *RM*3^•^ (**b**). Small yellow, gray, and red balls mark hydrogen, and common and target carbon atoms, respectively. The latter are additionally marked with small black balls. Larger green and red balls depict nitrogen and oxygen atoms. Magnified intermolecular junction areas (**c**). UHF AM1 calculations.

In the current study, the DTs represent the reagents and final products of the elementary reactions shown in Scheme 1. They involve a selected number of vinyl monomers M, namely, M1 (*N*-isopropylacrylamide—NIPA), M2 (*n*-butylacrylate—*n*BA), and M3 (styrene—St), equilibrium structures of which are shown in Figure 2a, as well as their monomer and oligomer radicals of n from 1 to 8. The free radical $R^{•}$ represents the 2-cyanopropyl radical, widely known as AIBN^•^. The virtual device in the current study is the CLUSTER-Z1 software [33,34] implementing the AM1 version of the semi-empirical unrestricted Hartree–Fock (UHF) approach [35,36]. The program has showed itself highly efficient concerning open-shell electronic systems such as fullerenes [37], graphene molecules [38], and stable radicals [39]. A short comment should be added concerning the choice of the virtual device. All quantum chemists recognize the radical nature of the FRP participants and discussions concerning the choice of the proper software to use have been at the forefront throughout the long development of the computational chemistry of polymers (see reviews [1,2,3,4,5,6,7,8,9,10,11,12,13,14,15,16,17,18] and references therein). The specifics of the QC description of stable radicals and open-shell electronic systems have been raised more than once in relation to virtual polymer science [40,41,42]. Nevertheless, today’s circumstances are such that the share of calculations using configurational interaction (CI) methods, even of the first level of complications, in the virtual polymers’ realm is relatively small. Justifying themselves by the long time and high financial costs of such calculations, practicing quantum chemists have chosen various versions of the DFT method, including unrestricted ones, as the main method of calculations. Unfortunately, DFT and UDFT do not work on systems with open electronic shells (see a detailed discussion of the problem elsewhere [43,44,45]). Accordingly, the numerous regularly appearing articles devoted to DFT-based virtual FRP do not move us along the path of truly significant results in this field of science. In the present work, this FRP quality feature is considered self-consistently for the first time. The spin-density algorithm for the step-by-step consideration of a series of successive intermolecular chemical addition reactions, generated in the depth of the UHF approximation [45,46], lays the foundations for this. This makes it possible to join the spin and electron features of open-shell radicalized species, thus distinctly determining the target atoms of the reagents subjected to the next attack of the attached atom or molecular group.

The IT product in the current study represents structural, thermodynamic, and kinetic parameters that, in terms of the universal energy graph, accompany the free-radical initiation of polymerization, a gradual transformation of the formed monomer radicals into oligomer ones in due course of the polymerization propagation followed by the growth of polymer chains, and the termination of the latter. The kinetic parameters are presented with the activation energy $E_{a}$, evaluated by using the EPS relation, previously checked by the reconstruction of a set of barrier profiles.

## 4. Initiation of Virtual Free-Radical Polymerization

### 4.1. Monomer Radical Generation

We begin our experiment on virtual the FRP of vinyl monomers by considering its first elementary reaction (1) related to the initiation. The largest systematic success in this area has been achieved when considering small molecules such as ethylene monomers and their derivatives in view of CI QC techniques [6,7]. When moving to larger monomers, the set of computational methods used narrows until DFT becomes the main method (see reviews [9,10,11,12,13,14,15,16,17,18] and references therein) that practically ignored the radical nature of the FRP participants. A selected free radical (see AIBN^•^ in Figure 2a) is to be attached to each of the chosen monomers, ensuring the formation of monomeric radicals. ${RM}^{•}$, which then subsequently lead a further propagation. To carry out this step, it is necessary to find out the most active atoms of the free radical and monomers thus ensuring the formation of the necessary intermolecular junction that should be a standard *sp*^3^C-C covalent bond.

To solve this part of the problem, we use a unique spin-density algorithm of atomic chemical susceptibility (ACS), the ACS algorithm below, which makes it possible to visualize the distribution of effectively unpaired electrons of radicalized species over their atoms [46]. Applied to AIBN^•^, this algorithm reveals the central carbon atom, marked by red in the figure, as the most active, characterizing it by the fractional number of unpaired electrons $N_{DA}$ = 0.807 *e*. The rest of the total number of radical unpaired electrons $N_{D}$ = 1.072 *e* is distributed over other carbon atoms (see Table 1). In monomers M1 and M2, both the total number of unpaired electrons $N_{D}$ and its fractions $N_{DA}$ on carbon atoms of vinyl groups are zero. This is because the lengths of the corresponding *sp*^2^C-C bonds are of 1.342 Å, which is much less than the critical value of the interatomic distance $R_{crit}^{C=C}$ = 1.395 Å in ethylene, starting from which *sp*^2^C-C bonds are radicalized [47]. Attaching radical AIBN^•^ to each atom of the vinyl bond of these monomers in turn reveals that a stable intermolecular junction is formed only in the case of its attachment to the atom of the methine (CH) group of vinyls in both cases. These atoms, marked by red in Figure 2a, are targets of the radical initiation of the corresponding monomers. In contrast to monomers M1 and M2, the structure of monomer M3 involves seven *sp*^2^C-C bonds. The position of the vinyl bond over the benzene ring greatly stimulates the generation of the unpaired electrons at the $R_{crit}^{C=C}$ by 0.5 Å less than the above value and provokes the delocalization of unpaired electrons over all carbon atoms, which leads to partial radicalization of all the *sp*^2^C-C bonds. The largest $N_{DA}$ of 0.106 *e* is located on the carbon atom of the methylene (CH_2_) group of the vinyl bond. The rest of the total number of $N_{D}$ = 0.747 *e* is distributed over the carbon atoms of the benzene ring by $N_{DA}$ of 0.093 *e* in average and over the rest one of the vinyl bond by $N_{DA}$ of 0.072 *e*. The attachment of radical AIBN^•^ to the benzene ring atom is highly unfavorable because of steric hindrance. Accordingly, the carbon atom of the vinyl CH_2_ group becomes the main target of the monomer for the radical initiation. Table 1 lists the main data related to the spin-density and energy parameters of the radical AIBN^•^ and the monomers.

Equilibrium structures of the intermolecular complexes related to the monomer radicals from *RM*1^•^ to *RM*3^•^, generated by the attachment of radical AIBN^•^ to the target atoms of the discussed monomers, are presented in Figure 2b. Actually, all of the bodies are radicals characterized by a nonzero $N_{D}$ (see Table 2). The main part of the values are concentrated on the carbon atoms of the methylene group of the monomer vinyl bond in the case of ${RM1}^{•}$ and ${RM2}^{•}$ (0.963 *e* in both case) and on the bond of the methine unit in the case of ${RM3}^{•}$ (0.670 *e*). The corresponding carbon atoms are shown in the figure in red and additionally marked with small black balls. Accordingly, the obtained ${RM1}^{•}$ and ${RM2}^{•}$ species are typical free radicals because of the sharp localization of the unpaired electrons on their target atoms. In contrast, 1.925 unpaired electrons of ${RM3}^{•}$ are distributed over all carbon atoms of the monomer radical, although with a clear preference to the atom of the methine unit with the largest $N_{DA}$. Thus, the carbon atoms of the methylene groups of the vinyl bonds of monomers M1 and M2, as well as that of the methine unit of monomer M3’s bond (see Figure 2c) are attacking atoms of the further FRP propagation under the leadership of monomer radicals ${RM1}^{•}$, ${RM2}^{•}$, and ${RM3}^{•}$, respectively.

To simplify further the description, we denote the carbon atoms, which form intermolecular junctions between the radical and monomers, such that C_1_ atoms refer to the target atom of the radical, C_2_—to the target atom of the vinyl group of monomers, and C_3_—to the remaining atom of the group. Thus, we obtain a possibility to present uniformly the atomic structure of the related intermolecular junctions as a combination of a single intermolecular *sp*^3^C_1_-C_2_ bond and double intramolecular *sp*^2^C_2_-C_3_ one. The unified first bond makes it possible to use it as a reaction coordinate when considering the barrier profile of the monomer radicals. In its turn, the second bond clearly reflects the positions of the previous and newly formed target atoms related to the vinyl bonds of the monomers. In contrast to the situation of the free monomers, the length of this bond is well above $R_{crit}^{C=C}$, mentioned earlier, thus providing significant $N_{DA}$ values of the new target atoms C_3_, as listed in Table 2. Evidently, this structure is typical for all vinyl polymers, differing by the hydrogen atom configuration (either *CH*_2_ or *CH*) around new targets only. As seen in the figure, the latter does not markedly affect the bond structure. In contrast, energy graphs are quite sensitive to the junction’s configuration.

Table 2 lists the spin-density and energy parameters related to the discussed monomer radicals from ${RM1}^{•}$ to ${RM3}^{•}$. The former is presented with the total and partial numbers of unpaired electrons, $N_{D}$ and $N_{DA}$, while the latter is a large collection of energy data related to the energy graphs accompanying the considered intermolecular elementary reaction. E(Y), E(X), and $E_{cpl}=E\left(X\right)-E(Y)$ represent the main parameters of the standard graphs determined directly at each calculation. As seen in the table, the $E_{cpl}$ distinguishes the ${RM1}^{•}$ and ${RM2}^{•}$ radicals from ${RM3}^{•}$ one by the absolute value for ${RM1}^{•}$ and ${RM2}^{•}$ being four-times reduced. The feature is qualitatively supported with the results of previous calculations [6,10,11,12,13,14,15], thus allowing separating monomers with a single *sp*^2^C-C vinyl bond from those with a set of the bonds. As will be shown later, this division on single-group and set-group monomers is fully approved concerning the FRP kinetics.

The next four columns are related to activation energies, among which $E_{ad}=E_{TS}-E(X)$ and $E_{aa}=E_{TS}-E(Y)$ denote the values related to either dissociation of the X product or association of the Y pair members, respectively. Since experimentally only the second values, usually marked as $E_{a}$, are determined, in what follows $E_{aa}$ will be substituted with $E_{a}$.

### 4.2. Transition State of Monomer Radicals

**Figure 3:**
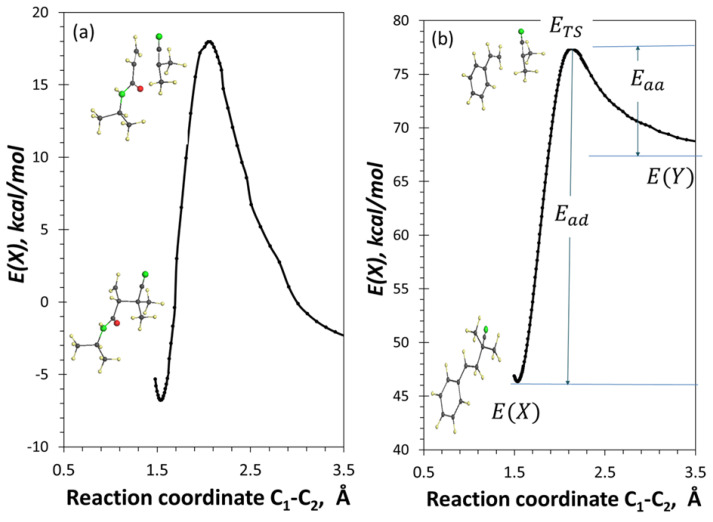
Barrier profiles of the dissociation of the monomer radicals ${RM1}^{•}$ (**a**) and ${RM3}^{•}$ (**b**). UHF AM1 calculations.

The transition state is a central point of the per-step-polymerization energy graph in Figure 1, indicating whether the dissociation and association of the polymerization participants has a barrier or is barrierless. Visualization of the graphs is possible when performing per-step-elongation QC calculations of the total energy E(X), related to each elementary reaction mentioned in Scheme 1, along a reaction coordinate (see examples of such procedure published elsewhere [32]). Concerning the reactions (1), evidently, intermolecular *sp*^3^C_1_-C_2_ bonds of the studied monomer radicals (see Figure 2c) are such coordinates in all the cases. This bond is kept fixed at each elongation step while all other coordinates are relaxed in looking for the total energy minimum. Figure 3 presents barrier profiles for the initiation steps of the FRP of monomers M1 and M3 caused by free radical AIBN^•^. The profiles were calculated in due course of the virtual dissociation of the monomer radicals ${RM1}^{•}$ and ${RM3}^{•}$, presented in Figure 2b. The basic spin-density and energy parameters related to the profiles are listed in Table 2. The insets in the figure present the equilibrium structures of the species in the ground and transition states. The energy graph of Figure 1, repeated in Figure 3b, gives a clear vision of the energy parameters under discussion.

As seen in the figure and shown in the table, the dissociation kinetics related to both monomer radicals should be quite similar. In contrast, the $E_{a}$s are very different, revealing a more than two-fold increase in ${RM1}^{•}$ compared to ${RM3}^{•}$. This feature is evidently connected with a difference between the $E_{cpl}$ values of the species discussed earlier and is of particular interest, indicating that free-radical initiation is kinetically more favorable for set-group vinyl polymers than for single-group ones. This conclusion correlates well with experimental data pointing to the same trend, as regularly observed concerning kinetic rate constants for initiation reactions [13,23,24]. As for other virtual data, the most deeply analyzed are related to methyl acrylate and styrene [13]. DFT-based data, obtained for transition state structures located adopting the synchronous transit-guided quasi-Newton method, are in agreement with ours for ${RM3}^{•}$, but differ for ${RM1}^{•}$. Taking into account the drastic difference in the computational techniques, it is not the difference in the $E_{a}$ for ${RM1}^{•}$, but the agreement of that for ${RM3}^{•}$ is the most exciting.

**Figure 4:**
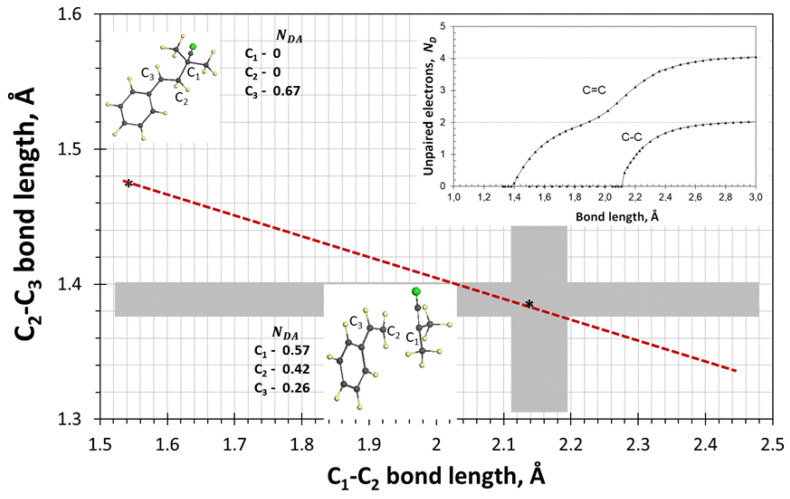
The interconnection between the *sp*^2^C=C and *sp*^3^C-C bonds constituting the intermolecular junction of the ${RM3}^{•}$ monomer radical. See the junction determination in Figure 2c. Inset presents the dissociation of the *sp*^2^C=C bond of ethylene and *sp*^3^C-C bond of ethane. UHF AM1 calculations.

An attentive analysis of the transition states in both cases reveals a particular common behavior in spite of drastically different kinetics. First, attention is drawn to the value of the reaction coordinate corresponding to the maximum of the barrier profile. As seen in Table 3, the values are located around 2.100 Å in both cases. This is close to $R_{crit}^{C-C}≅$ 2.110 Å that determines the start of breaking the *sp*^3^C-C bond in ethane [47]. As it turns out, $R_{crit}^{C-C}$ deviates rather markedly, responding to the changing of the atomic configuration around the breaking bond [38] and providing quite a marked dispersion. We evaluate this dispersion, related to the intermolecular junctions under study, as ~0.10 Å. This fact forces one to look attentively on the studied junctions that are the united configuration of the discussed single intermolecular *sp*^3^C-C bond and the intramolecular *sp*^2^C-C bond of the monomers’ vinyl units. Obviously, these bonds are tightly interconnected, which is revealed as a close interrelation of their lengths, as seen in Figure 4 where the red broken line depicts the dissociation of the *sp*^3^C-C bond, which starts at the (x,y) point (1.535,1.471), indicating that the junction consists of a standard *sp*^3^C-C bond and elongated *sp*^2^C-C one. Because of the elongation, the double bond is radicalized [47], thus providing unpaired electrons of the total number $N_{D}$ = 1.925 *e*, which causes the generation of the junction target atom C_3_ with $N_{DA}$ = 0.67 *e*. The $N_{D}$ value is in agreement with a general law that governs a gradual radicalization of the *sp*^2^C-C bonds, presented as an inset in Figure 4. Similarly, to *sp*^3^C-C bonds, the start of the *sp*^2^C-C bond radicalization occurs at $R_{crit}^{C=C}≅$ 1.395 Å for ethylene that is dispersed as well, thus reflecting influence of the bond’s surrounding. For the current case, the dispersion is evaluated as ~0.02 Å. The dispersion zones for $R_{crit}$ values for both bonds are presented with vertical and horizontal light gray bands, related to the *sp*^3^- and *sp*^2^-bonds, respectively.

The ${RM3}^{•}$ dissociation is completed at point (4.203,1.342) (not shown), going through the transition state at point (2.129,1.387). As seen in the figure, the point is located in the region where the lengths of both bonds are close to critical. Judging by the $N_{DA}$ values at this point and referring to the inset, we can conclude that the transition state corresponds to the radicalization of both bonds. In this case, the *sp*^2^C-C bond is only at the very beginning of its long way to breaking, while the started radicalization of the *sp*^3^C-C bond means the beginning of its breaking. The sharp threshold-like transition of the energy graphs around $R_{crit}^{C-C}$ is provided by the spin density of the species and marks either the exit of the spin density from zero values (dissociation) or the spin density zeroing during the formation of a standard single bond (association). It can be assumed that it is the spin nature of the transition state control that makes it so universally significant for any chemical reaction [31]. It is obvious that the transition state described above, based on a single *sp*^3^C-C bond, will always occur at the bond length of 2.10 ± 0.10 Å. This conclusion can be enlarged by stating that the transition state of any elementary reaction occurs at the $R_{crit}$ length of the covalent bond that is the reaction coordinate of the case.

## 5. First-Step Propagation of Virtual Free-Radical Polymerization—The Generation of Dimer Radicals

**Figure 5:**
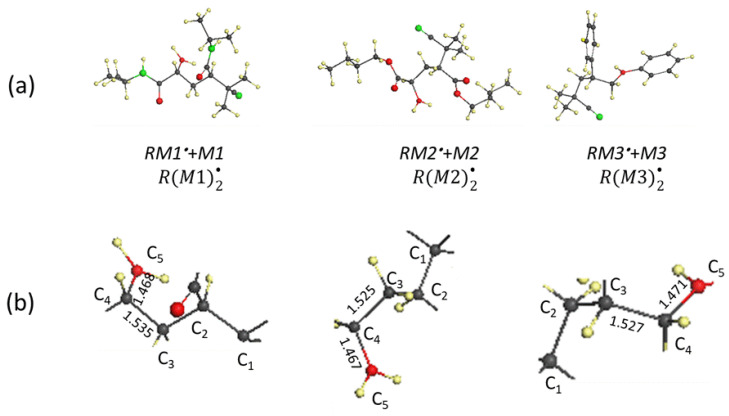
Dimer radicals based on vinyl *sp*^2^C=C bond. Equilibrium structures of dimer radicals ${R(M1)}_{2}^{•},\;{R(M2)}_{2}^{•},\;and\;{R(M3)}_{2}^{•}$ (**a**). Small yellow, gray, and red balls mark hydrogen, and common and target carbon atoms, respectively. The latter are additionally marked with small black balls. Larger green and red balls depict nitrogen and oxygen atoms. Magnified intermolecular junction areas (**b**). UHF AM1 calculations.

The first-step in the FRP propagation, the first elementary reaction of type (2) in Scheme 1, represents the generation of dimer radicals from monomer radicals following the general scheme ${RM}^{•}$ + $M={R(M)}_{2}^{•}.$ Equilibrium structures of the dimer radicals related to monomers from M1 to M3 are shown in Figure 5a. The corresponding intermolecular two-part junctions are presented in Figure 5b. As seen in the figure, the first parts of the junctions, formed by carbon atoms from C_1_ to C_3_ and playing the governing role in the case of the monomer radicals, are now extended to involving atoms C_4_ and C_5_. This part, discussed in the previous section, is changed because of the transformation of the *sp*^2^C_2_-C_3_ bond into a standard *sp*^3^C_2_-C_3_ one. The newly formed intermolecular *sp*^3^C_3_-C_4_ bond provides the connection of the monomer with the relevant monomer radical, while the intramolecular *sp*^2^C_4_-C_5_ bond takes the baton of the transfer of radical properties. The corresponding spin-density parameters of the dimer radicals are listed in Table 2. As seen in the table, these values practically repeat those related to the monomer radicals, thus indicating a strong similarity of the intermolecular junctions from the spin viewpoint. The C_5_ carbon atoms play the role of newly formed targets, aimed at holding a further polymerization. As for the energy parameters, in contrast to the monomer radicals, $E_{cpl}$ are more homogeneous, thus reflecting a common origin of the second part of the intermolecular junctions.

**Figure 6:**
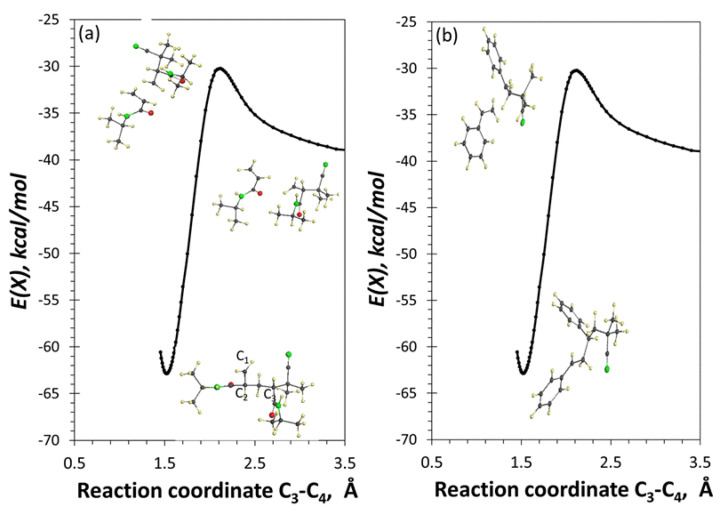
Barrier profiles of the dissociation of the dimer radicals ${R(M1)}_{2}^{•}\left(a\right)\;and\;{R(M3)}_{2}^{•}$ (**b**). UHF AM1 calculations. See the junction determination in Figure 5b.

Repeating the consideration of activation energies, performed previously for the monomer radicals ${RM1}^{•}$ and ${RM3}^{•}$, Figure 6 presents barrier profiles of the dissociation of dimer radicals ${R(M1)}_{2}^{•}\;and\;{R(M3)}_{2}^{•}$ along the evident reaction coordinate that is the *sp*^3^C_3_-C_4_ bond. Similarly to the previous case, the energy graphs are completed with equilibrium structures of the species and with their structures in the transition state as well as with a dissociated structure in the case of ${R(M1)}_{2}^{•}$. As seen in the figure, both graphs look quite similar with respect to $E_{ad}$. This is a clear consequence of the close similarity of the $E_{cpl}$ values of both radicals. The $E_{a}$ values are more sensitive to the difference in $E_{cpl}$, which still retains a rather similar value for both species. These values correlate with experimentally determined values quite well [48,49], as well with other DFT-based data [13,50].

The main characteristics of the transition states of both dimer radicals are given in Table 3. As seen in the table, the latter are quite similar to those of the monomer radicals, thus supporting the previously made conclusion that the transition state is governed by the spin-density properties of the current intermolecular junctions formed by a pair of the *sp*^2^C_4_-C_5_ and *sp*^3^C_3_-C_4_ bonds. Since, as will be shown in the next section, the intermolecular junction at each subsequent step of polymerization is governed by the same combination of covalent bonds, the data listed in Table 3 can be extended to other oligomer radicals, starting from trimer ones.

The presence of pairs of barrier profiles, related to the monomer and dimer radicals of monomers M1 and M3, makes it possible to generate EPS Equation (2), related to elementary reactions of types (1) and (2) in Scheme 1. Using the data for $E_{cpl}$ and $E_{a}$ from Table 2, we obtain the following relations

(3) $$E_{a}=22.65+0.63E_{cpl}\;\left(\mathrm{ER}\;\mathrm{type}\;1\right)$$

(4) $$E_{a}=20.03+0.48E_{cpl}\;\left(\mathrm{ER}\;\mathrm{type}\;2\right)$$

As it turns out, parameters M and α for both elementary reactions are quite similar, which strengthens confidence in the reliability of their use for determining $E_{a}$ values of other vinyl radicals. The latter, thus obtained for monomer and dimer radicals of monomer M2, are listed in Table 2.

## 6. Vinyl Oligomer Radicals of Free-Radical Polymerization

**Figure 7:**
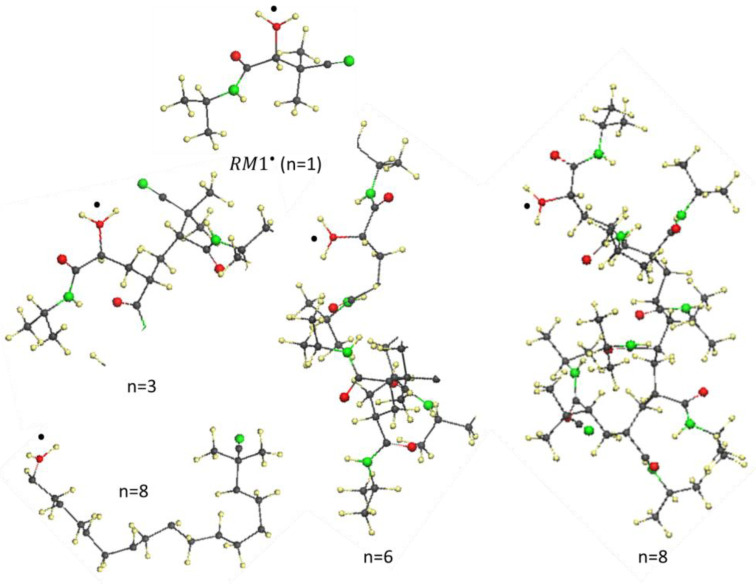
Equilibrium structures of oligomer radicals ${R(M1)}_{n}^{•}$ for n = 1, 3, 6, and 8. Small yellow, gray, and red balls mark hydrogen, and common and target carbon atoms, respectively. The latter are additionally marked with small black balls. Larger green and red balls depict nitrogen and oxygen atoms. UHF AM1 calculations.

Let us continue the generation of oligomer radicals following the subsequent steps of the reaction ${RM}^{•}+\left(n-1\right)M={RM_{n}^{•}}^{}$ for *n* = 1, 2, 3, 4, *…* up to *n* = 8, 6, 7 for monomers M1,M2, and M3, respectively. The radicals ${R(M1)}_{n}^{•}$ for *n =* 3, 6, and 8 are presented in Figure 7. The corresponding members with *n =* 4, 5, and 7 are shown in Figure S1. The collection in Figure 7 is accompanied by a final ‘clothesline’ that unites nine intermolecular junctions, eight of which are formed of two *sp*^3^C-C bonds each, while the last one involves a pair of *sp*^3^- and *sp*^2^-bonds, the latter holding the newly formed target atoms. The line of atoms is accompanied by alternate methine and methylene groups. Attention is drawn to the bizarre weaving pattern of the oligomeric chain that differs drastically from regular chains straightened into one line, which abound in the presentation of linear polymers in articles and monographs. This circumstance is caused by the fact that each new addition of a monomer to the chain is accompanied by a change in the electronic configuration of the target carbon atom of the previous intermolecular junction, leading to the *sp*^2^→*sp*^3^ transformation of the corresponding bond. The dramatic change in the valence angles under conditions of the obligatory avoidance of steric hindrance is extremely unfavorable for the rectilinear configuration of the clothesline, thus twisting it in such a complicated way. Table 4 lists the main spin-density and energy parameters of the designed oligomer radicals, starting from trimer ones.

**Figure 8:**
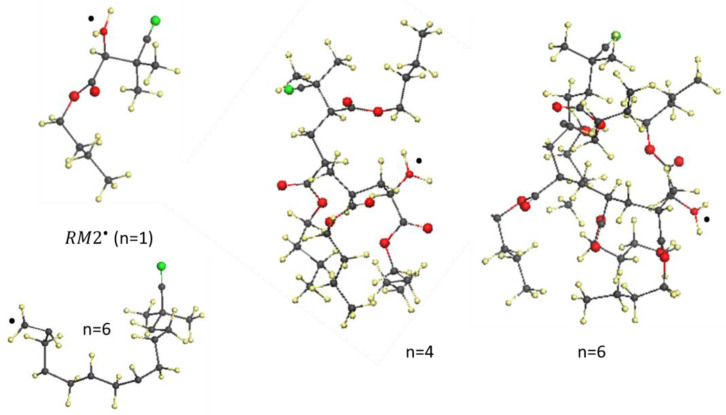
Equilibrium structures of oligomer radicals ${R(M2)}_{n}^{•}$ for n = 1, 4, and 6. Small yellow, gray, and red balls mark hydrogen, and common and target carbon atoms, respectively. The latter are additionally marked with small black balls. Larger green and red balls depict nitrogen and oxygen atoms. UHF AM1 calculations.

**Figure 9:**
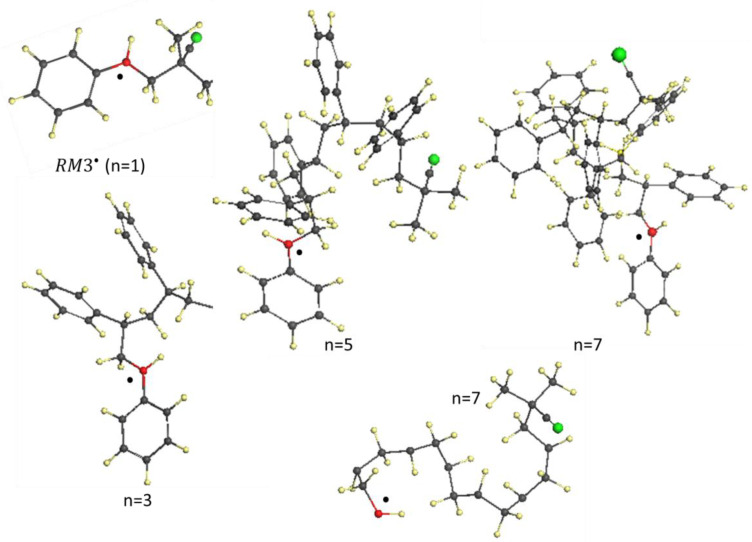
Equilibrium structures of oligomer radicals ${R(M3)}_{n}^{•}$ for n = 1, 3, 5, and 7. Small yellow, gray, and red balls mark hydrogen, and common and target carbon atoms, respectively. The latter are additionally marked with small black balls. Larger green balls depict nitrogen atoms. UHF AM1 calculations.

The radicals ${R(M2)}_{n}^{•}$ for *n =* 4 and 6 alongside with the relevant clothesline are presented in Figure 8. The corresponding members with *n =* 2, 3, and 5 are shown in Figure S2. As seen from the figures, what is shown convincingly confirms on a qualitative level everything that was said above for oligomer radicals ${R(M1)}_{n}^{•}$, allowing us to confidently extend the identified polymer chain growth algorithm to all vinyl monomers, which are various alkenes with a single vinyl group. However, as follows from Figure 9 and Figure S3, which represent a complete collection of oligomer radicals ${R(M3)}_{n}^{•}$, monomers that involve sets of *sp*^2^C-C bonds also obey this algorithm. Thus, oligomeric radicals, presented in Figure 7, Figure 8, Figure 9 and Figures S1–S3, give a complete picture of the algorithm for the radical polymerization of vinyl monomers at the stage of linear chain formation.

A full collection of the main spin-density and energy parameters of the designed oligomer radicals, starting from trimer ones, are presented in Table 4. The wide range of values of $E_{cpl}$ presented in the table stimulates the use of the previously obtained ESR from Equation (4) for determining the $E_{a}$ values that concern the growth of the relevant chains. As can be seen from the table, in the ${R(M1)}_{n}^{•}\;case$, the deviations of the smallest and largest $E_{a}$ values from the average value of 7.97 kcal/mol are 21% and 12%, respectively. For ${R(M2)}_{n}^{•}$, the same values make up the series 9.32 kcal/mol and 10% and 19%. For ${R(M3)}_{n}^{•}$, this series has the following form: 10.97 kcal/mol, 44%, and 50%. The percentage values determine the degree of stability of the propagation rate constant $k_{p}$ along the corresponding chains. As follows from the above data, in chains ${R(M1)}_{n}^{•}$ and ${R(M2)}_{n}^{•}$, the stability is quite high in contrast to ${R(M3)}_{n}^{•}$, which might indicate the greater role of steric aspects in the latter case.

## 7. Termination of the Polymer Chain Growth

**Figure 10:**
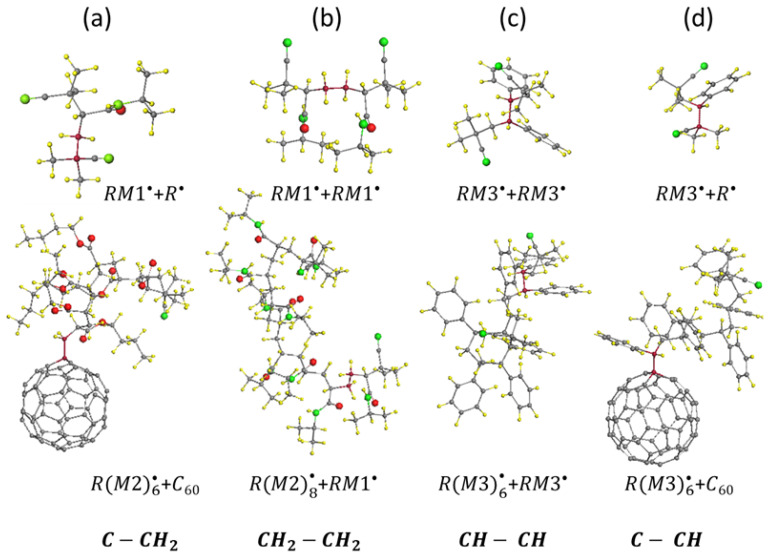
Equilibrium structures of products $P_{n}$ of elementary reaction of type (3) providing the termination of the polymer chain growth. ${{RM}^{•}+R}^{•}$ and ${R(M)}_{n}^{•}$ + ${RM}^{•}$ products for single-group (**a**) and set-group (**d**) vinyl monomers, respectively. The same, but for ${RM}^{•}+C_{60}$ and ${R(M)}_{n}^{•}$ + ${RM}^{•}$ products (**b**,**c**), respectively. Small yellow, gray, and red balls mark hydrogen, and common and target carbon atoms, respectively. Larger green and red balls depict nitrogen and oxygen atoms. Chemical formulae of the intermolecular junctions’ *sp*^3^C-C bonds are presented in the last row. UHF AM1 calculations.

The third stage of linear polymerization, concerning the termination of the polymer chain growth and related to elementary reactions of type (3), proved to be the most complex. As previously, termination is governed by intermolecular interactions that couple the growing chain with other available species from the polymerization reactor. Evidently, the reactor content is very rich and is gradually replenished as polymerization progresses, starting with free radicals and extending over monomer, dimer, and other oligomer radicals. All these species can be coupled with the current chain, thus terminating its growth. Examples of stopping of this kind are widely described in a large number of monographs. Starting to analyze this stage virtually, we are faced with two serious problems. The first is the already mentioned richness of molecular participants in the reactor. The second concerns the great role of steric aspects. It is obvious that one should speak of a chain at the level of oligomer radicals with a sufficiently large n. As can be seen from Figure 7, Figure 8 and Figure 9, as n increases, the chain structure becomes more and more complex. Since the intermolecular interaction is carried out with the participation of vinyl bond atoms in the majority of cases, the structural adaptation of the participants with respect to each other encounters many obstacles. Figure 10 presents examples of intermolecular junctions, characteristic for elementary reactions of type (3) related to FRP-involved vinyl oligomer radicals and achieved after overcoming steric hindrances. The name of each junction is indicated under each reaction product. The configurations of the resulting junctions are clearly visible in the simplest intermolecular products, presented in the first row of the figure and concerning the interaction of the radical monomer with the free radical or itself. As in the previous cases, the monomer radicals of the single-group and set-group monomers behave differently, generating C-CH_2_ (a) and C-CH (d) junctions, when presented with a standard *sp*^3^C-C bond, respectively. Two red-marked carbon atoms match the junctions in each case. The same junctions are observed if the radical ${AIDN}^{•}$ is replaced with a more complex one, for example, with C_60_ fullerene, and the monomer is substituted with an oligomer ${R(M2)}_{6}^{•}$ (a) or ${R(M3)}_{6}^{•}$ (b).

The second type of intermolecular contact occurs when monomer and oligomer radicals, belonging to the same monomer, are coupled to each other. As can be seen from the example of the two monomer radicals in the first row, the atomic structure of the intermolecular junction is an *sp*^3^C-C bond between either two methylene (b) or methine (c) groups for single- and set-group monomers, respectively. The same contact structure is retained when one of the monomer radicals is replaced by an oligomer one, such as ${R(M1)}_{8}^{•}$ in the first case and ${R(M3)}_{6}^{•}$ in the second.

The intermolecular complexes shown in the figure reflect only a small number of the potential structures in the reactor. They are characterized by a significant scatter in the $E_{cpl}$ values from 4 to 45 kcal/mol and, accordingly, in the $E_{a}$ values, in the range from 23 to 2 kcal/mol. A significant part of these energies’ dispersion should be attributed to steric effects. However, there is no doubt that prediction of the winners paths in these complex processes is difficult, and, therefore, the development of a special algorithm is required to foresee this path. The solution to this problem requires further research using not only the DT concept, but also quantum computing.

Fullerene C_60_, as a replacement for the free radical ${AIDN}^{•}$, was not chosen in this work by chance. Since their discovery, both fullerenes C_60_ and C_70_ have been often considered as perfect templates for the chemical adsorption of large molecules [51,52]. Exciting images of star-like derivatives with straight molecular rays can be found in many articles and monographs devoted to the molecular chemistry of fullerenes’ derivatives [37,53]. These observations have led to the foundation of the idea of a star-like molecular-ray configuration of polymer chains terminated with fullerenes [54,55,56,57,58]. However, as seen in Figure 10, the polymer chains are not straight lines and, in contrast, present rather cumbersome intricate open structures. The resulting fullerene derivatives are quite stable and are characterized by significant coupling energies (−3.9 kcal/mol and −34.6 kcal/mol for $C_{60}{R(M2)}_{6}$ and $C_{60}{R(M3)}_{6}$, respectively). As can be seen from Figure 10, the space required for a comfortable arrangement of oligomer radicals in both cases is big, so that the simultaneous accommodation of five or more radicals on the fullerene is impossible. Stereometric analysis shows that no more than four such radicals can be attached to an empty fullerene C_60_ if steric hindrance is to be avoided.

## 8. Conclusions

The discussion of virtual FRP proposed in this article rests on the presentation of polymerization as a chain reaction subdivided into successive elementary reactions. This vision allows the thermodynamics and kinetics of the reaction to be considered in the format of standard energy graphs. The implementation of the concept in the current study is based on the following conceptual grounds. First, the concept of digital twins, which is most suitable not only for the consideration of each such reaction, but also provides for their comparative analysis, highlighting reactions of the same type carried out under identical conditions. With a self-consistent approach to the choice of research objects (DTs), the virtual device, and the desired virtual intellectual product, a virtual experiment becomes a legitimized analogue of a real one, endowed with broad power. Over 60 DTs were considered in the current study. With such possibilities, we have carried out a rather full review of the entire cycle of the FRP of vinyl polymers related to its nanoscale aspect.

The second conceptual issue concerns the dominant role of intermolecular interactions, which determine the inevitability of the all-with-all interaction between participants in the multicomponent basin of a polymerization reactor. Polymer formation is seen as a way to win the competition. The uniqueness of the controlling interaction gives rise to the uniqueness of the general algorithm of the polymerization process, based on the universal energy graphs that describe the energy component of the process at all stages.

The spin-density algorithm of the UHF approximation, which makes it possible to obtain a numerical description of effectively unpaired electrons, is the third conceptual basis of the above consideration. This algorithm application first solves the problem of the adequate consideration of the open electronic shells of the radical reaction participants, and then confidently determines the positions of target carbon atoms within the initial and intermediate reagents, and establishes the governing role of spins in the localization of the transition state in the reaction coordinate system.

The fourth conceptual element of the consideration results from the universality of the energy graphs mentioned above, which makes it possible to use barrier profiles, or the dependences of the total energy of the current polymer fragment on the reaction coordinate, for direct determination of the activation energy $E_{a}$ of the current polymerization step.

The close relationship between $E_{cpl}$ and $E_{a}$ is the basis of the fifth conceptual element, expressed in terms of the Evans–Polanyi–Semenov relation. Found experimentally, this relationship was directly confirmed in the present work and is proposed for a reliable estimation of $E_{a}$ for the entire line of consecutive chain fragments.

Following these principles, the virtual FRP of vinyl monomers can be described as follows. The field of competence of quantum-chemical consideration extends to linear chains of sequential oligomeric radicals with the number of chain links n and the total length L of these radicals’ clothesline, determined by the computing capabilities of the digital device, i.e., by the programs and computers used. In the present work, the number of links reached eight, and the corresponding length L was ~30 Å. This turned out to be possible due to the high efficiency of the semi-empirical UHF approximation that forms the foundation of the virtual device used.

The considered polymer chains represent oligomer radicals ${R(M)}_{n}^{•}$ that are the product of intermolecular interactions between the free radical and n-oligomer. The digital twins’ design of these radicals is based on three monomers and a varying number of monomer units in the chain from one to eight. The choice of monomers was subordinated to the number of vinyl *sp*^2^C-C bonds in the monomer. So, alkene monomers M1 and M2 represent species with a single vinyl group. In contrast, the structure of the M3 monomer (styrene) contains seven such bonds. The paper shows that the spin-density and energy characteristics of these two types of monomers are indeed different.

The obtained thermodynamic characteristics of the considered DTs, which cover structural data and energy parameters concerning the ground state of the molecules, revealed the following common characteristics related to polymer chains and their members.

- Oligomer radicals ${R(M)}_{n}^{•}$, starting with monomer one, constitute the successive links of the monomers;
- The first link *RM*^•^ is produced in the stage of the polymerization initiation, while all the rest occur in due course during the gradual polymerization propagation;
- All the studied vinyl monomers form stable oligomer radicals characterized with a significant $E_{cpl}$;
- Spin-density and energy parameters can distinguish monomers by the number of the involved vinyl groups, thus dividing them into single-group and set-group monomers;
- The structural composition of all the n-oligomer radicals is subordinated to the spin-density algorithm of formation and is configured around a basic ‘clothesline’ consisting of n intermolecular junctions, n − 1 of which are composed of one intermolecular and one intramolecular *sp*^3^C-C bond, accompanied by methine and methylene units, while the remaining currently working one involves an elongated intramolecular *sp*^2^C-C bond, thus providing a target for radical attack;
- A consequent *sp*^2^→*sp*^3^ transformation of the covalent C-C bonds, accompanying the clothesline elongation, combined with the resistance to emerging steric hindrances, has resulted in a bizarre, non-straight line image of the clothesline.

The kinetics of the oligomer radicals’ generation is considered in terms of the activation energy $E_{a}$ corresponding to the association of the relevant starting reagents. Universal energy graphs, forming the foundation of intermolecular interaction energetics and resting on the basic energy parameters $E\left(X\right)\;and\;E(Y)$, $E_{cpl}$, $E_{TS}$, and $E_{a}$, form the consideration grounds. The analysis of the obtained data from this viewpoint has revealed the following.

- Characterizing the transition state by $E_{TS}$ is the central point allowing the direct determination of $E_{a}$;
- $E_{TS}$ was determined when constructing barrier profiles for two pairs of oligomer radicals involving *RM*1^•^ and *RM*3^•^ as well as ${R(M1)}_{2}^{•}$ and ${R(M3)}_{2}^{•}$ that represent elementary reactions of types (1) and (2), respectively;
- The obtained profiles clearly distinguish single-group and set-group monomers, thus pointing to a different kinetics of the latter;
- On the background of this difference, the transition state reveals a particular common feature related to the identical position of the profile maximum on the reaction coordinate, which is the intermolecular *sp*^3^C-C bond of the current junction, at $R_{crit}^{C-C}≅$ 2.1 Å in all the cases;
- Simultaneously, with a critical behavior of this *sp*^3^ bond, the length of the junction intramolecular *sp*^2^C-C bond takes a value of 1.378 Å, which is $R_{crit}^{C=C}$ that fixes the beginning of the radicalization of the *sp*^2^ bonds under elongation;
- Framing the energy parameter $E_{TS}$ with two critical values, related to covalent *sp*^3^ and *sp*^2^ bonds, simultaneously indicates the spin-determining nature of the transition state of the vinyl oligomer radicals;
- The obtained $E_{a}$ data, determined as the difference between $E_{TS}$ and $E\left(Y\right)$, combined with the $E_{cpl}$ data of these oligomer radicals, allows the restoration of the EPS relations connecting the two sets of values for both pairs, attributing them to two different elementary reactions;
- The obtained EPS relation, concerning the elementary reaction of type (2), laid the foundations for obtaining $E_{a}$ values for all the rest of the oligomer radicals;
- The $E_{a}$-based kinetic behavior of single-group and set-group oligomer radicals is different with respect to the initiation and propagation stages of FRP: the former is greatly favorable for the set-group ones, while the latter is slightly more favorable for the single-group ones.
- The discussed trends are related to the initiation and propagation stages of free-radical polymerization, while revealing similar trends for the termination of polymer chains is still waiting for a particular algorithm.

To summarize this part on activation energy in radical addition reactions, we can compare the obtained data with the following important factors which were reported to play a governing role in the barrier formation [7,59].

*Reaction Exothermicity*. The larger the $\left|∆H\right|$, in other words, the larger the $\left|E_{cpl}\right|$, the lower the $E_{a}$. This factor takes place at all stages of the current study.

*The Singlet–Triplet Energy Gap,* $E_{ST}$*, of the Alkene*. The smaller the $E_{ST}$, the lower the $E_{a}$. The $E_{ST}$ values of the considered monomers are given in Table 1. As seen from the table, the monomers’ properties correlate well with this factor.

*Polar Effects*. These effects lower $E_{a}$ when $\left(I\left(R\right)-EA(A)\right)$ or $\left(I\left(A\right)-EA(R)\right)$ < 9–9.5 eV (or 7–8 eV when including experimental data). The calculated data on the ionization potentials (I) and electron affinity (EA) of all the studied alkenes and radicals fit the pointed values, thus pointing to a possible ignoring of polar effects in the FRP of vinyl monomers.

## Figures and Tables

**Table 1:** Spin-density and energy (kcal/mol) parameters of the virtual FRP participants.

Monomer	$N_{D}$, *e*	$N_{DA}$, *e* ^(1)^	$l_{bond}$, Å ^(2)^	$E_{tot}$	$E_{ST}$ ^(3)^
R (${AIDN}^{•})$	1.072	0.807	-	30.73	-
M1 (NIPA)	0	0	1.342	−31.85	67.52
M2 (*n* BA)	0	0	1.342	−89.14	61.56
M3 (St)	0.747	0.107	1.344	38.14	24.31

^(1)^ The atomic chemical susceptibility of the carbon target atom. ^(2)^ The length of the vinyl *sp*^2^C-C bond. ^(3)^ $E_{ST}=E_{T}-E_{S}$ is the energy gap between singlet and triplet states of the molecules.

**Table 2:** Spin-density and energy (kcal/mol) parameters of the FRP-involved vinyl monomer and dimer radicals.

Radical	$N_{D}$	$N_{DA}$ ^(1)^	E(Y)	E(X)	$E_{cpl}$	$E_{ad}$	$E_{aa}\equiv E_{a}$	$E_{aexp}$	$E_{acalc}$
${RM1}^{•}$	1.024	0.963	−1.12	−6.79	−5.67	24.76	19.09		10.0 [13]
${RM2}^{•}$	1.024	0.963	−58.41	−64.36	−5.85		18.96 (3) ^(2)^		
${RM3}^{•}$	1.925	0.67	68.87	46.36	−22.51	31.01	8.5		8.9 [13]
${R(M1)}_{2}^{•}$	1.025	0.96	−38.64	−62.9	−24.26	32.65	8.39	4.37 [48]	6.26 [48]
${R(M2)}_{2}^{•}$	1.024	0.965	−153.5	−176.34	−22.84		9.07 (4) ^(2)^		
${R(M3)}_{2}^{•}$	1.928	0.668	84.5	67.89	−16.61	28.67	12.06	7.79 [49]	8.32 [50] 11.30 [13]

^(1)^ The values are related to the newly formed target atoms C_3_ and C_5_ of the monomer radicals (Figure 2c) and dimer ones (see Section 5), respectively. ^(2)^ Numbers in parentheses indicate the number of the EPS equation used to determine $E_{a}$ (see text).

**Table 3:** Spin-density, energy, and structure parameters of the transition states of the FRP-involved vinyl monomer and dimer radicals.

Radical	$N_{D}$, *e*	$N_{DA}$, *e*	$E_{TS}$, kcal/mol	$l_{bond}$, Å ^(1)^	$l_{bond}$, Å ^(2)^
		$C_{1}$; $C_{2}$; $C_{3}$		$C_{1}$-$C_{2}$	$C_{2}$-$C_{3}$
${RM1}^{•}$	1.424	0.54; 0.55; 0.19	17.97	2.069	1.383
${RM3}^{•}$	2.369	0.57; 0.42; 0.26	77.37	2.129	1.387
		$C_{2}$; $C_{3}$; $C_{4}$		$C_{3}$-$C_{4}$	$C_{4}$-$C_{5}$
${R(M1)}_{2}^{•}$	1.412	0.67; 0.49; 0.19	−30.25	2.123	1.373
${R(M3)}_{2}^{•}$	3.157	0.54; 0.41; 0.27	96.56	2.107	1.388

^(1)^ *sp*^3^C-C bonds of the current junctions. ^(2)^ Vinyl *sp*^2^C=C bonds of the current junctions (see Figure 2c and Section 5).

**Table 4:** Spin-density and energy (kcal/mol) parameters of the FRP-involved oligomer radicals of vinyls.

Radical	$N_{D}$, *e*	$N_{DA}$, *e*	E(X)	$E_{cpl}$	$E_{a}$
${R(M1)}_{3}^{•}$	1.024	0.964	−118.26	−23.51	7.74
${R(M1)}_{4}^{•}$	1.024	0.964	−174.16	−24.05	8.49
${R(M1)}_{5}^{•}$	1.024	0.96	−233.93	−27.92	6.3
${R(M1)}_{6}^{•}$	1.024	0.961	−289.62	−23.84	8.59
${R(M1)}_{7}^{•}$	1.024	0.965	−346.97	−25.5	7.79
${R(M1)}_{8}^{•}$	1.024	0.965	−401.97	−23.15	8.92
${R(M2)}_{3}^{•}$	1.024	0.962	−289.71	−24.23	8.4
${R(M2)}_{4}^{•}$	1.024	0.963	−402.76	−23.91	8.55
${R(M2)}_{5}^{•}$	1.024	0.961	−514.52	−22.62	9.17
${R(M2)}_{6}^{•}$	1.024	0.964	−622.18	−18.52	11.14
${R(M3)}_{3}^{•}$	1.963	0.668	86.11	−19.92	10.47
${R(M3)}_{4}^{•}$	2.146	0.67	105.35	−18.9	10.96
${R(M3)}_{5}^{•}$	2.16	0.669	124.26	−19.23	10.8
${R(M3)}_{6}^{•}$	2.376	0.667	155.02	−7.38	16.49
${R(M3)}_{7}^{•}$	3.073	0.667	164.16	−29	6.11

## Data Availability

Any data or material that support the findings of this study can be made available by the corresponding author upon request.

## Supplementary Materials

The following supporting information can be downloaded at: https://www.mdpi.com/article/10.3390/polym15142999/s1, Figure S1: Equilibrium structures of oligomer radicals ${R(M1)}_{n}^{•}$ for *n* = 1, 2, 4, 5 and 7. Small yellow, gray and red balls mark hydrogen, common and target carbon atoms, respectively. The latter are additionally indicated by small black balls. Larger green and red balls depict nitrogen and oxygen atoms. UHF AM1 calculations; Figure S2: Equilibrium structures of oligomer radicals ${R(M2)}_{n}^{•}$ for *n* = 1, 2, 3 and 5. Small yellow, gray and red balls mark hydrogen, common and target carbon atoms, respectively. The latter are additionally indicated by small black balls. Larger green and red balls depict nitrogen and oxygen atoms. UHF AM1 calculations; Figure S3: Equilibrium structures of oligomer radicals ${R(M3)}_{n}^{•}$ for *n* = 1, 2, 4, and 6. Small yellow, gray and red balls mark hydrogen, common and target carbon atoms, respectively. The latter are additionally indicated by small black balls. Larger green balls depict nitrogen atoms. UHF AM1 calculations.
