# Supplementary material for: Virtual Free-Radical Polymerization of Vinyl Monomers in View of Digital Twins
Source: Polymers (Basel). 2023 Jul 10;15(14):2999. doi: 10.3390/polym15142999 (PMC10383948; doi:10.3390/polym15142999)
Supplement: Supplementary file 1 [file polymers-15-02999-s001.zip › polymers-2441152-supplementary.pdf]

Virtual Free-Radical Polymerization of Vinyl Monomers in View of Digital Twins

Elena F. Sheka

Institute of Physical Researches and Technology,
Peoples' Friendship University of Russia (RUDN University), 117198 Moscow, Russia

sheka@icp.ac.ru

SUPPORTING MATERIALS

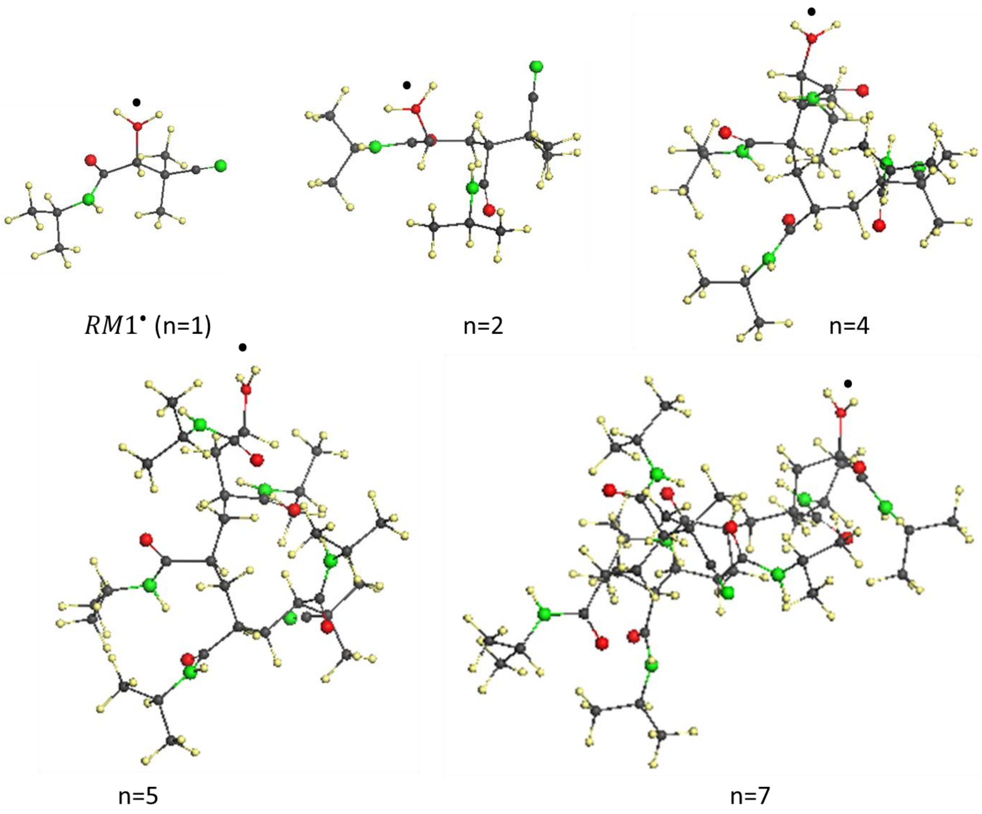

Figure S1. Equilibrium structures of oligomer radicals $R(M1)_n^{\bullet}$ for n= 1, 2, 4, 5 and 7. Small yellow, gray and red balls mark hydrogen, common and target carbon atoms, respectively. The latter are additionally indicated by small black balls. Larger green and red balls depict nitrogen and oxygen atoms. UHF AM1 calculations.

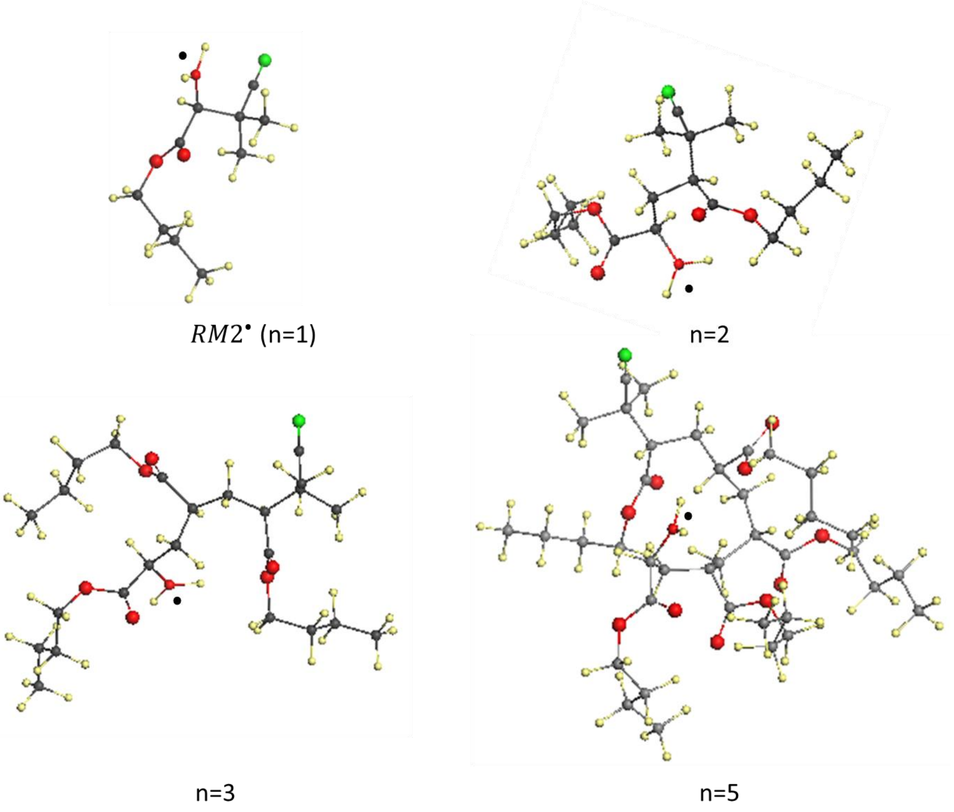

Figure S2. Equilibrium structures of oligomer radicals $R(M2)_n^{\bullet}$ for n= 1, 2, 3 and 5. Small yellow, gray and red balls mark hydrogen, common and target carbon atoms, respectively. The latter are additionally indicated by small black balls. Larger green and red balls depict nitrogen and oxygen atoms. UHF AM1 calculations.

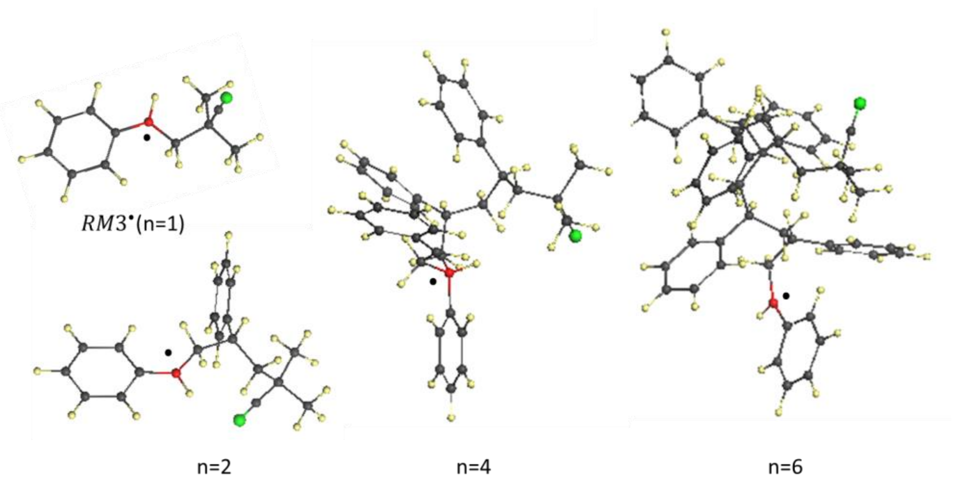

Figure S3. Equilibrium structures of oligomer radicals $R(M3)_n^{\bullet}$ for n= 1, 2, 4, and 6. Small yellow, gray and red balls mark hydrogen, common and target carbon atoms, respectively. The latter are additionally indicated by small black balls. Larger green balls depict nitrogen atoms. UHF AM1 calculations.
